# Internal biliary drainage for isolated posterior segmental biliary obstruction: a case report

**DOI:** 10.1186/s13256-018-1699-7

**Published:** 2018-06-04

**Authors:** Hideki Izumi, Hisamichi Yoshii, Daiki Yokoyama, Shuji Uda, Rin Abe, Masaya Mukai, Eiji Nomura, Hiroyuki Ito, Takahiko Mine, Tomohiro Matsumoto, Terumitsu Hasebe, Hiroyasu Makuuchi

**Affiliations:** 1 Department of Gastrointestinal Surgery, Tokai University Hachioji Hospital, 1838 Ishikawa, Hachioji, Tokyo, 192-0032 Japan; 2 Department of Internal Medicine, Tokai University Hachioji Hospital, 1838 Ishikawa, Hachioji, Tokyo, 192-0032 Japan; 3 Department of Diagnostic Radiology, Tokai University Hachioji Hospital, 1838 Ishikawa, Hachioji, Tokyo, 192-0032 Japan

**Keywords:** Bile duct injury, Internal bile duct drainage, Pancreaticoduodenectomy

## Abstract

**Background:**

Biliary system anatomical abnormalities can be preoperatively detected on magnetic resonance imaging; therefore, some presume that the number of bile duct injuries should decline. However, once a bile duct injury occurs, repair may be difficult. There are various ways to repair bile duct injuries, but successful repair may be exceptionally difficult.

**Case presentation:**

A 72-year-old Japanese man underwent a pancreaticoduodenectomy due to a diagnosis of middle bile duct cancer. We had a complication of an isolated posterior segmental biliary obstruction when pancreaticoduodenectomy was performed. We conducted a drip infusion cholecystocholangiography-computed tomography test to determine the positional relationship between his bile duct and elevated jejunum. To secure the bile duct we punctured the bile duct under computed tomography guidance, and the hepaticojejunal anastomosis site was visualized by inserting an endoscope. We vibrated the bile duct wall by inserting a guide wire through a puncture needle and verified the vibrations with the endoscope. We observed a partially compressed elevated jejunal wall upon guide wire insertion; therefore, we could verify a puncture needle penetration into the elevated jejunum by endoscope on insertion. We also successfully inserted an 8.5-Fr pigtail catheter into the elevated jejunum. We removed all drains after percutaneously inserting an uncovered metallic stent. Our patient’s subsequent clinical course was unremarkable. He visits our institution as an out-patient and has had no stent occlusion even after 6 months.

**Conclusions:**

When repairing bile duct injuries, it is important to accurately determine the positional relationships between the injured bile duct and the surrounding organs.

## Background

Pancreaticoduodenectomy is one of the most difficult surgeries. The incidence of postoperative complications can be as high as 30–50%, depending on the case [1–4]. Pancreatic fluid leakage is frequently observed [5, 6], and various complications such as intraabdominal abscess, hemorrhage, bile leakage, and bile duct injury may occur. Because biliary system anatomical abnormalities can be preoperatively detected on magnetic resonance imaging (MRI), some presume that the number of bile duct injuries should decline. However, once a bile duct injury occurs, repair may be difficult. We report on a case of successful establishment of internal bile duct drainage, into the elevated jejunum, against an isolated posterior segmental biliary obstruction after pancreaticoduodenectomy. We discuss therapeutic approaches for bile duct injury repair as well as our method of establishing internal biliary drainage.

## Case presentation

**Fig. 1:**
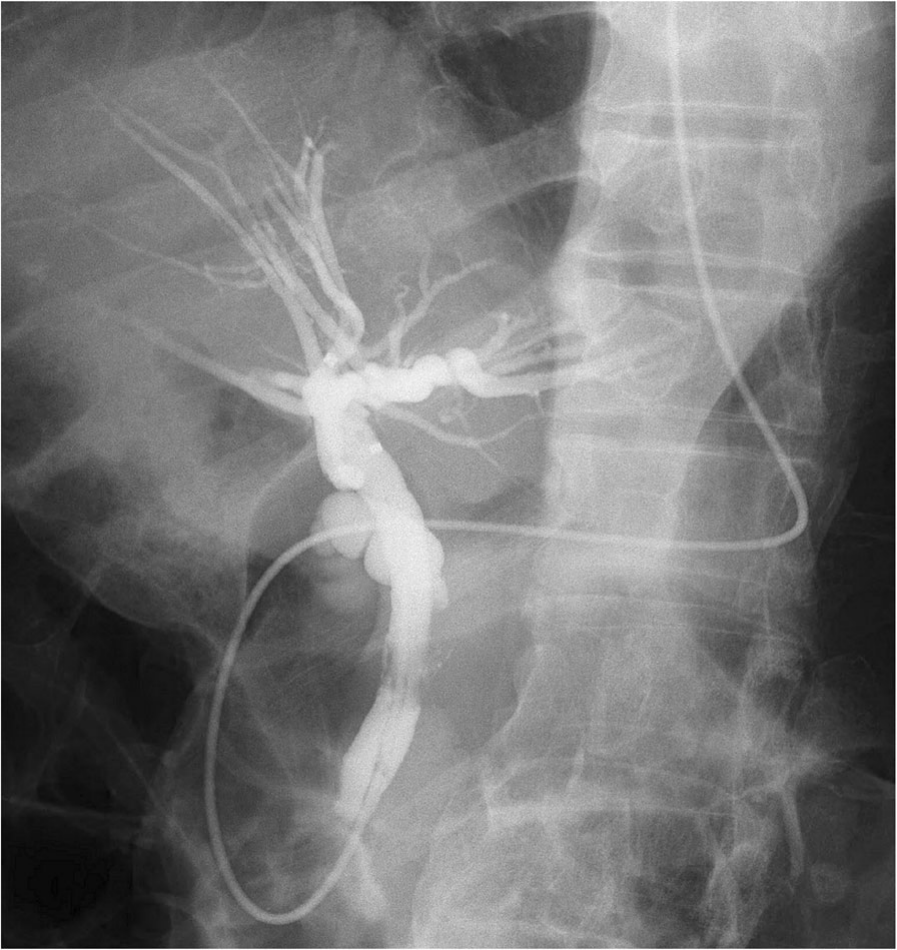
Preoperative endoscopic retrograde cholangiopancreatography image

**Fig. 2:**
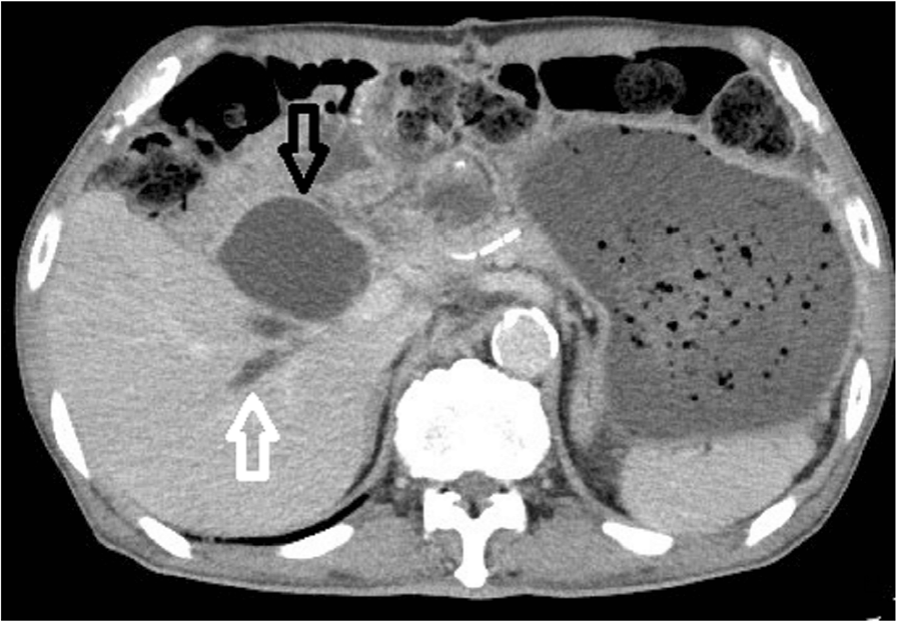
Postoperative computed tomography image: The *black arrow* indicates a tumor, suggestive of bile leakage, and the *white arrow* indicates a dilated posterior bile duct

**Fig. 3:**
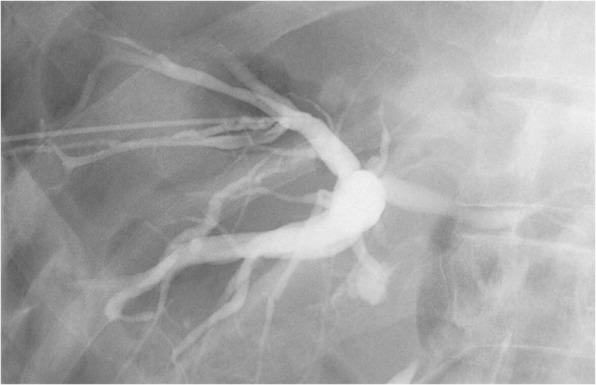
Percutaneous transhepatic biliary drainage contrast radiography. Only bile ducts in the posterior segment were visualized without a bile leakage

**Fig. 4:**
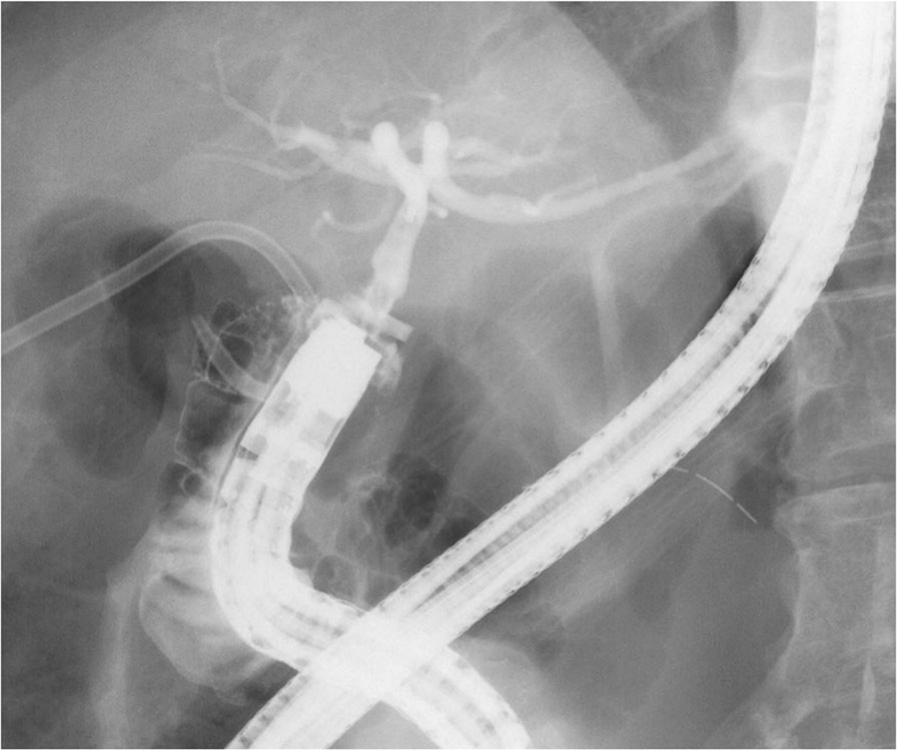
Endoscopic contrast radiography. Only the anterior segmental branch and left branch were visualized; the posterior segmental branch was not visualized

Our patient was a healthy 72-year-old Japanese man, with an unremarkable previous medical history. He was referred to our institution due to jaundice and impaired hepatic function found during a health examination. We observed stenosis in the middle bile duct on a preoperative endoscopic retrograde cholangiopancreatography (ERCP) image (Fig. 1), whereas class V adenocarcinoma was detected by biliary abrasive cytology. The preoperative image indicated low bifurcation in the posterior segmental branch. A pancreaticoduodenectomy was conducted due to the diagnosis of middle bile duct cancer. Because our patient had no post-surgical complaints, even given mildly increased inflammation, he was discharged on postoperative day 22. However, we found increased inflammation on blood withdrawal when he visited our institution on postoperative day 30. On computed tomography (CT) we observed abscess formation with suspected bile leakage around the hepaticojejunal site and posterior segmental bile duct dilatation (Fig. 2). We initially completed percutaneous transhepatic biliary drainage (PTBD). During contrast radiography with PTBD, only the posterior segmental branch was visualized, but there was no bile leakage into the elevated jejunum (Fig. 3). Later, we completed contrast radiography from the hepaticojejunal anastomosis site with the use of an endoscope, and only the anterior segmental branch and left branch were visualized (Fig. 4). Thus, we concluded the damage was on the low bifurcation in the posterior segmental branch. Bile (approximately 250 ml/day) was discharged by PTBD on consecutive days.

**Fig. 5:**
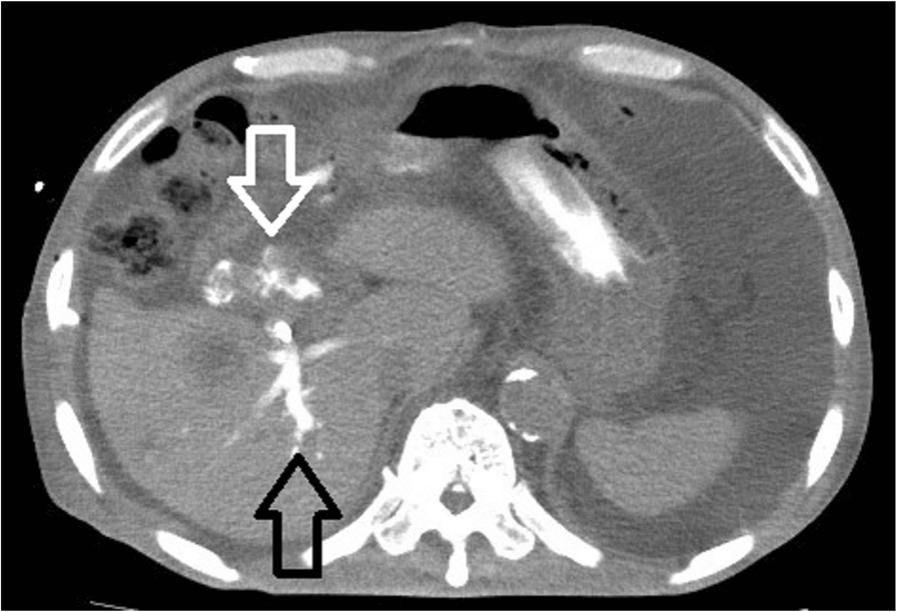
Drip infusion cholecystocholangiography-computed tomography contrast agent (*white arrow*) discharged into the elevated jejunum from the anterior segmental branch and left branch. The *black arrow* indicates the posterior segmental branch

We conducted a drip infusion cholecystocholangiography (DIC)-CT test to determine the positional relationship between bile duct and elevated jejunum. We found contrast agent discharged into the elevated jejunum from the anterior segmental branch and left branch (Fig. 5; white arrow). We dorsally visualized the bile duct in the isolated posterior segmental branch (Fig. 5; black arrow). From the DIC-CT test, we at that time detected an unclear positional relation between elevated jejunum and posterior segmental branch. We determined that there was no intrusion of other organs between the elevated jejunum and the bile duct. Consequently, percutaneous transhepatic internal drainage of the posterior isolated bile duct, to the elevated jejunum, could be conducted.

**Fig. 6:**
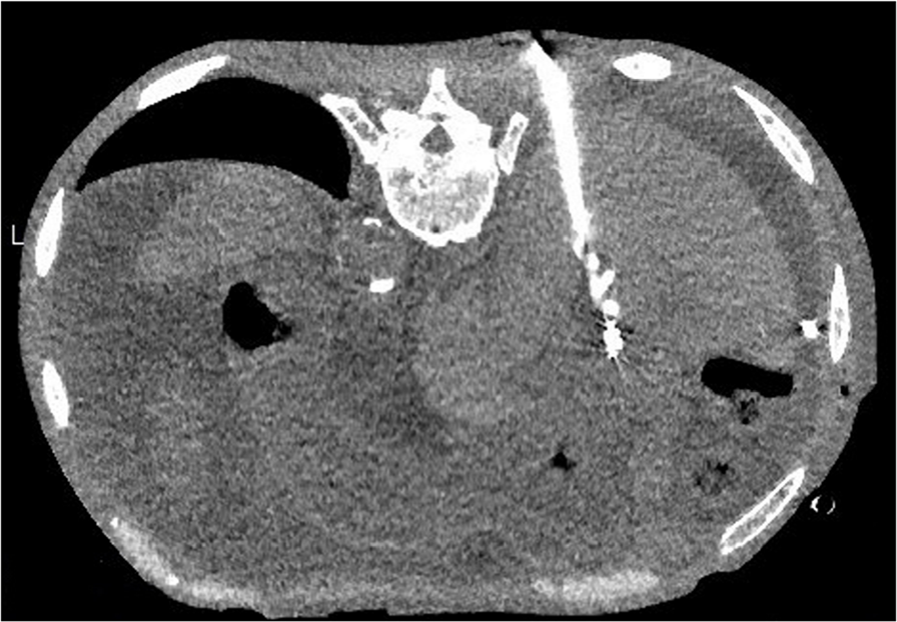
Puncture with computed tomography guidance

**Fig. 7:**
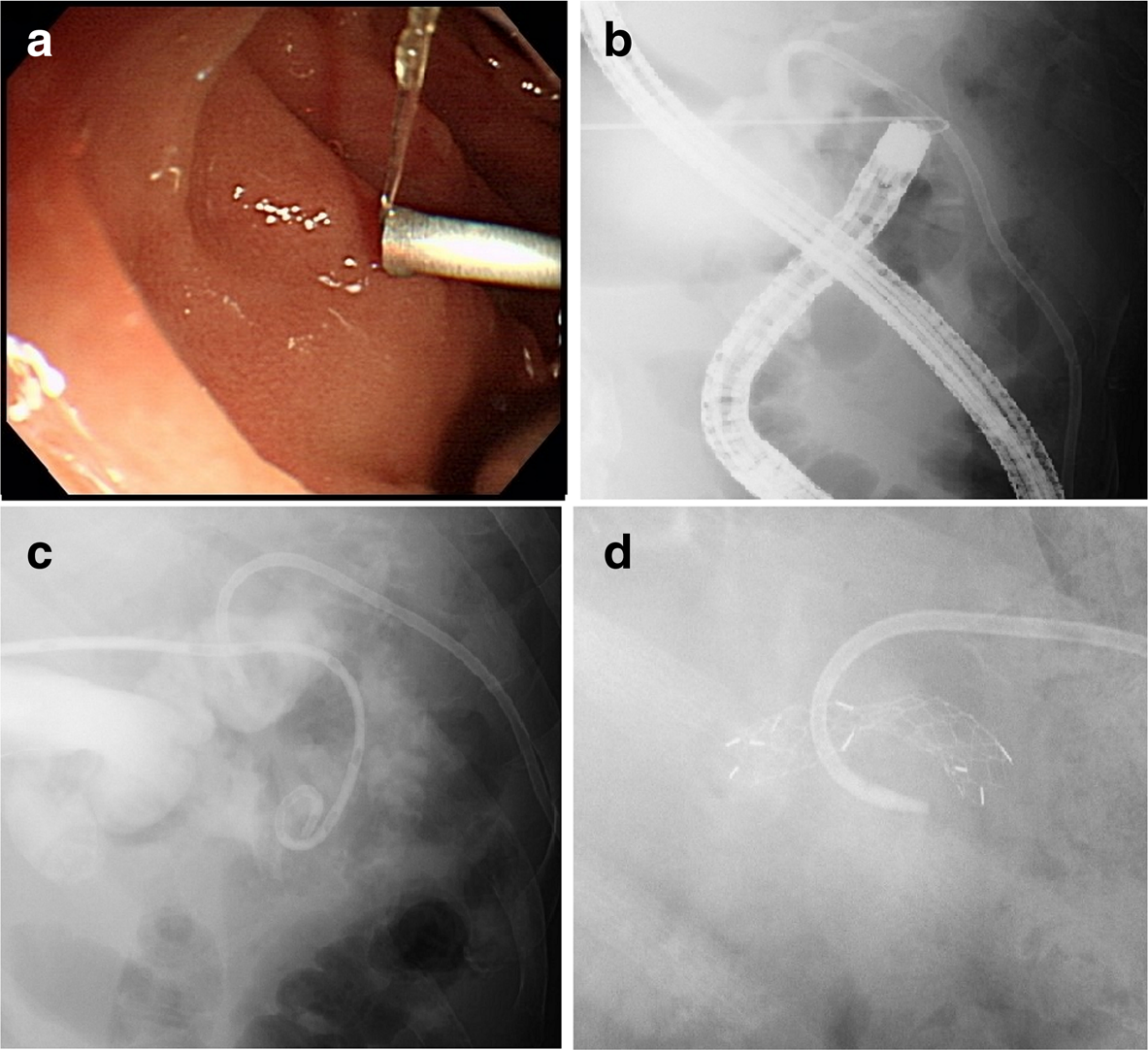
**a** Puncture needle penetration into elevated jejunum. **b** X-ray at the time of penetration of puncturing needle into the elevated jejunum. **c** 8.5-Fr pigtail catheter was inserted into the elevated jejunum. **d** Uncovered metallic stent (5 cm 10 mm) was inserted percutaneously

To secure the bile duct, we made a puncture in the bile duct (Fig. 5; black arrow) under CT guidance (Fig. 6), and visualized the hepaticojejunal anastomosis site by inserting an endoscope. We vibrated the bile duct wall by inserting a guide wire through a puncture needle, and verified the vibrations with the endoscope. We found a partially compressed elevated jejunal wall upon guide wire insertion; therefore, we could verify a puncture needle penetration into the elevated jejunum by endoscope on insertion (Fig. 7a, b). We also successfully inserted an 8.5-Fr pigtail catheter into the elevated jejunum (Fig. 7c). We removed all drains after percutaneously inserting an uncovered metallic stent (5 cm 10 mm; Fig. 7d). Our patient’s subsequent clinical course was unremarkable, and he visits our institution on an out-patient basis, without stent occlusion even after 6 months.

## Discussion

In patients who undergo hepato-biliary-pancreatic surgery, postoperative bile leakage can be a frequent complication. Although the frequency of bile duct injury leakage after hepatectomy has been reported [7–9], there is no report of bile duct injury after pancreaticoduodenectomy.

Hardening and adhesion by an anomalous cystic duct, segmental bile duct branch anatomy, or pathological inflammation can cause bile duct injury [10]. Preoperative detection of bile duct branch abnormalities is important to help reduce complications. Unfortunately, our patient experienced bile duct damage and dissection because we failed to check the preceding bifurcation in the posterior segmental branch, although noticing it before surgery. According to Kitami *et al.* [11], preceding bifurcation in the posterior segmental branch requires careful attention due to its incidence (4.5–7.5%). The treatment method for bile duct injury can vary depending on the degree of injury or time of diagnosis [10].

There are two main treatments for bile duct injury. The first approach is to reduce biliary excretion. In these cases, hepatectomy may be the most reliable approach [12]. However, it should not be selected in cases involving large invasion. Alternatively, anhydrous ethanol injection [13, 14] and selective portal vein embolization [15, 16] can be considered. Becker *et al.* [17] and Majeed *et al*. [18] reported that the use of anhydrous ethanol for the biliary system was effective for ablating the gall bladder and cystic duct, but Kyokane *et al*. [13] reported an indication relating to the intrahepatic bile duct. First, they confirmed the safety of the procedure during animal experiments, and then conducted biliary ablation only for the hepatic lateral segment B2, against bile duct leakage, after hepatectomy for gallbladder cancer. Hepatic atrophy, at the site of biliary ablation, and hepatic enlargement, at the non-injection site, were confirmed [13, 14]. Selective percutaneous transhepatic portal embolization may be effective for promoting hepatic atrophy and fibrosis, as well as reducing bile production [15, 16].

The second method is to establish a new biliary excretion route. Bile duct reanastomosis [10] and magnet compression anastomosis (Yamanouchi’s method) [19] are examples of these type of approaches. Bile duct reanastomosis can be difficult to perform due to the thin diameter of the bile duct, particularly where anastomotic stricture may also be present. Furthermore, identification of damaged site for the segmental bile duct branch can be extremely difficult during a repeat surgery due to adhesion with surrounding tissue. This surgery can be extremely difficult; therefore, it is not easily adopted [20, 21]. Yamanouchi’s method naturally forms an anastomosis by placing powerful magnets that make the targeted intestinal lumens attract each other [19]. Initially, the approach was indicated for anastomosis of intestinal tract-to-intestinal tract at the time of intestinal obstruction, but it was recently adapted for anastomosis of the bile duct-to-intestinal tract, where it was successful [22]. Because the approach is less invasive and features a high success rate, it might be a worthwhile procedure for many patients.

## Conclusions

We report a successful case of treating isolated posterior bile duct injury with new internal drainage. Bile duct injury repair methods are varied; however, it is important to accurately determine the positional relationships between the injured bile duct and surrounding organs.
